# Current Evidence for Developmental, Structural, and Functional Brain Defects following Prenatal Radiation Exposure

**DOI:** 10.1155/2016/1243527

**Published:** 2016-06-12

**Authors:** Tine Verreet, Mieke Verslegers, Roel Quintens, Sarah Baatout, Mohammed A. Benotmane

**Affiliations:** Radiobiology Unit, Laboratory of Molecular and Cellular Biology, Institute for Environment, Health and Safety, Belgian Nuclear Research Centre, SCK•CEN, 2400 Mol, Belgium

## Abstract

Ionizing radiation is omnipresent. We are continuously exposed to natural (e.g., radon and cosmic) and man-made radiation sources, including those from industry but especially from the medical sector. The increasing use of medical radiation modalities, in particular those employing low-dose radiation such as CT scans, raises concerns regarding the effects of cumulative exposure doses and the inappropriate utilization of these imaging techniques. One of the major goals in the radioprotection field is to better understand the potential health risk posed to the unborn child after radiation exposure to the pregnant mother, of which the first convincing evidence came from epidemiological studies on* in utero* exposed atomic bomb survivors. In the following years, animal models have proven to be an essential tool to further characterize brain developmental defects and consequent functional deficits. However, the identification of a possible dose threshold is far from complete and a sound link between early defects and persistent anomalies has not yet been established. This review provides an overview of the current knowledge on brain developmental and persistent defects resulting from* in utero* radiation exposure and addresses the many questions that still remain to be answered.

## 1. Introduction

The health benefits of the use of radiation in medical practice are nowadays widely acknowledged. Nevertheless, also possible negative outcomes for human health after ionizing radiation exposure are recognized. For instance, the risks for pregnant women, more precisely for their embryo/fetus to develop structural and functional brain defects, have been described based on epidemiological studies from atomic bomb survivors. These observations are particularly relevant in the case of radiotherapy treatment or medical imaging during pregnancy, which often results in abortion, delay of maternal therapy, or preterm delivery when women were diagnosed with cancer during pregnancy [1]. Hence, treatment of pregnant women with cancer is clearly suboptimal. Furthermore, the use of medical radiation modalities employing low doses, such as CT scans, has increased tremendously over the past decades [2]. The advantages of this increasing use of low doses in the hospital are obvious, but a major concern exists regarding the cumulative exposure doses of repetitive tests and the inappropriate utilization of these imaging techniques [3, 4]. In all, the benefits of current medical practice can still be improved for pregnant women and their unborn child, for which a better understanding of radiation effects, especially in the low-dose range, is imperative.

**Figure 1:**
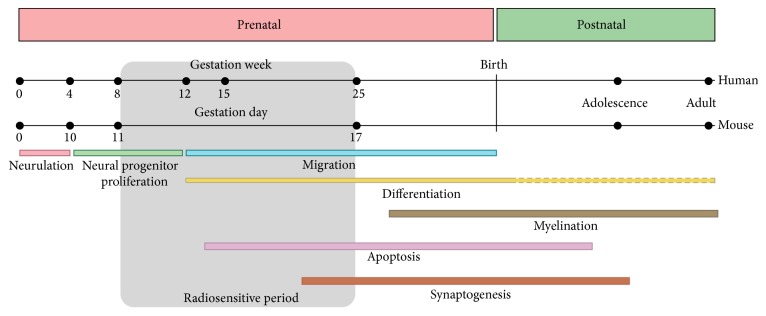
Milestones of human and mouse neocortical development, with indication of the most radiosensitive period. Modified from [9].

The human developing brain is extremely sensitive to radiation exposure, which is especially the case when exposure occurs within a specific developmental time-window, that is, between weeks 8 and 25 of gestation [5]. This period is characterized by specific coordinated developmental processes and corresponds to embryonic days 11 (E11) to 17 in mice (see Figure 1). In most cases, the mouse has been the experimental model of choice to investigate radiation-induced defects to the brain, but the high vulnerability of the fetal brain to environmental insults has been recognized in many other mammalian species, including primates and other rodents [6–8]. Research on prenatal radiation-induced brain defects has mainly focused on the developing neocortex, of which the developmental hallmarks are depicted in Figure 1. However, persistent brain defects are presumed to result from a concerted action of different brain regions, going beyond the neocortex and including other major brain structures such as the hippocampus, cerebellum, and striatum. Most of these regions undergo neurogenesis during the gestational period [9], with the exception of the cerebellum and hippocampal dentate granule cells that display crucial developmental and neurogenic events during the early postnatal period and further [8]. Thus, to understand the full extent of developmental aberrations to the brain following irradiation, we need to be aware of the necessity to investigate all brain regions that might be affected by irradiation, which is currently still lacking. It is only then that we can identify the causative factors responsible for the observed brain damage in prenatally exposed survivors of the atomic bombings in Hiroshima and Nagasaki, which manifested as an increase in the frequency of mental retardation, lower IQ and school performance, and unprovoked seizures [5]. Concomitantly, this knowledge would be highly beneficial for the medical sector, where the use of ionizing radiation is common standard and increasing constantly and where we are facing many uncertainties on the exact impact of (repeated) low-dose exposures. Here, we review the current knowledge, but also the concerns and limitations, regarding early and persistent defects to the brain after* in utero* exposure to low- and moderate-to-high doses of irradiation.

## 2. Epidemiological Evidence for Prenatal Radiation-Induced Noncancer Effects

In 1929, Goldstein and Murphy reported on mental retardation and microcephaly resulting from prenatal radiation exposure, as revealed from 38 case reports of children born to mothers that received pelvic radiotherapy [10]. Decades later, this awareness was further strengthened and quantitative data were provided through the follow-up of the health of atomic bomb survivors, primarily performed and published by Otake and Schull [5]. Their study involved 1500 individuals exposed* in utero* to the radioactive fallout of the atomic bombs in Hiroshima and Nagasaki (mainly *γ*-radiation). Apart from an excess cancer risk [11], a higher incidence in generalized growth retardation and microcephaly, mental disability, and seizures, as well as a decreased school performance and scoring on intelligence tests, was observed [5]. These defects were all relatively linearly dose-dependent, with an increased risk for mental retardation of 43% and a decline of 25–29 points in IQ values per Gy [12]. No dose threshold was proposed for these observations, except for mental retardation, for which symptoms were detected at doses as low as 0.06 to 0.31 Gy [13]. Important to note from these studies is that the developing brain is particularly sensitive to irradiation when exposure occurred between weeks 8 and 15 of pregnancy and to a lesser extent between weeks 16 and 25 [12, 14]. Hence, the brain appears especially vulnerable to radiation during the period characterized by a massive neuron production and differentiation/migration (Figure 1).

The fallout of the Chernobyl accident in 1986 has exposed many people to radioiodine (^131^I) and radiocaesium (^137^Cs). Also here, prenatally irradiated subjects were followed over time, but findings are much less consistent and are subject to debate [15, 16]. This might be due to the fact that people in the surrounding areas of the catastrophe were exposed to relatively low doses (between 0.01 and 0.25 Sv), with, for instance, an important Norwegian cohort receiving doses less than 0.10 Sv. Other limitations of these epidemiological studies were the potential confounding variables that could not be taken into account, the lack of accurate dose measures per individual, and the fact that cohorts were considerably smaller than those of the atomic bomb survivors [17, 18]. Nevertheless, an increased occurrence of mental retardation and decrease in (verbal) IQ scores could be noted in children and adolescents* in utero* exposed [18–21]. Neuropsychiatric problems were also reported but might as well be associated with the mother's health and stress [20]. Noteworthy, consistent with previous findings, the radiosensitive period to develop such anomalies involved weeks 8 to 25 [19]. This was further corroborated by a recent study involving a cohort of prenatally exposed children of whom the mothers were exposed to diagnostic X-ray pelvimetry during late pregnancy, and thus beyond the peak of neurogenesis. During this late stage, no association of irradiation with school performance was found, although this might also be due to the low doses of radiation received by the fetus (0.5–9 mGy) in this study [22].

In all, it is evident that, above a certain threshold, irradiation during the gestational period hampers normal brain development and functioning during later life when exposure occurred during the peak of neuronal expansion and differentiation. While experimental findings can vary depending on the study, most credible results were obtained by epidemiological investigations in atomic bomb survivors. Yet the lack of knowledge about the underlying causes warranted a more in-depth research involving* in vitro* and* in vivo* animal models, which will be elaborately discussed in the following paragraphs.

## 3. Experimental Evidence from Animal Models

In search for defects that occur shortly after prenatal radiation exposure and that might account for long-lasting cognitive defects as observed in human cohorts, animal studies have been proven a highly valuable tool [23]. The rodent brain is a widely used model system, given the obvious advantages of working with small rodents, the ease to genetically modify the mouse genome, and most importantly the high similarity in brain development, architecture, and interconnectivity when compared to humans [24].

### 3.1. Early Prenatal Irradiation Effects

**Figure 2:**
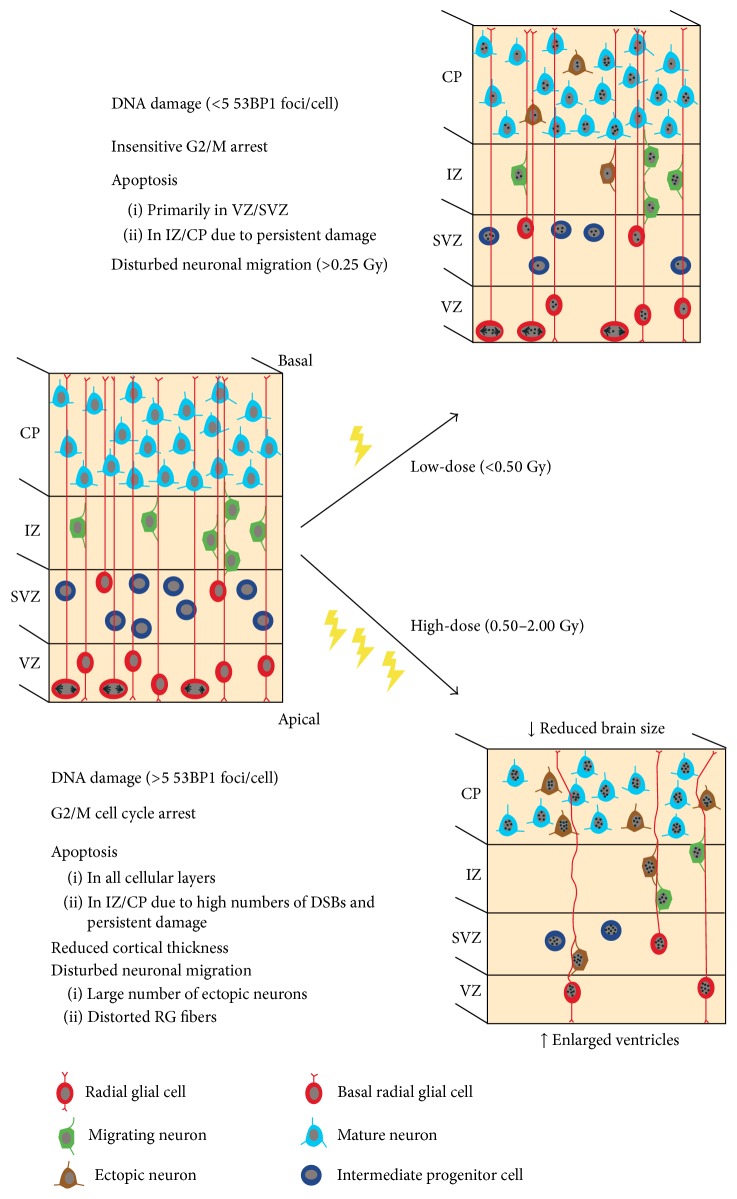
Overview of current knowledge regarding early low- and moderate-to-high-dose prenatal irradiation-induced events. 53BP1: P53 binding protein 1, CP: cortical plate, DSB: double-strand break, Gy: Gray, IZ: intermediate zone, RG: radial glial cell, SVZ: subventricular zone, and VZ: ventricular zone.

As a defense mechanism to radiation exposure, a series of biochemical pathways are activated to promote cell survival while maintaining genetic integrity. Yet not all cells are equally sensitive to radiation. Proliferating cells are considered much more vulnerable to radiation-induced damage because they require correct and intact DNA for their progeny. In postmitotic cells on the other hand, the integrity of their transcribed genes is considered crucial rather than that of their whole genome [25]. This is exemplified by the fact that the immature rodent brain is much more sensitive to radiation stress as opposed to the juvenile or adult brain that contains very few proliferating cells [26]. During the radiosensitive embryonic period (Figure 1), neuronal precursor cells appear even more susceptible to radiation damage than proliferating cells in other embryonic tissues [27]. It is therefore imperative to closely examine the central nervous system in order to gain knowledge about prenatal radiation-induced health risks. A schematic overview of brain developmental processes that are altered after prenatal radiation exposure and that are described in detail in the following paragraphs is depicted in Figure 2.

#### 3.1.1. Radiation-Induced DSBs and Cell Cycle Arrest

Differences in radiosensitivity between neural stem/progenitor cells and postmitotic neurons may be related to differences in the radiation-induced DNA damage response (DDR) between these cell types. DNA damage and the production of reactive oxygen species (ROS) and reactive nitrogen species (RNS) is a general hallmark after irradiation in mammalian cells. This is for example shown in cultured rat embryonic cells irradiated with a high dose of 2.0 Gy, generating an excess of ROS and RNS within few hours after radiation [28]. The high sensitivity of the developing brain to oxidative stress is thought to be due to its low concentrations of antioxidants, its particular lipid configuration of the cell membranes, its high rate of oxygen consumption, and the large proportion of sensitive immature cells [29]. Of note, however, the limited antioxidative capacity is also characteristic of postmitotic neurons in the developing brain [30], thus seeming to be in contrast with the proposed sensitivity of neural precursors to radiation-induced DNA damage [31, 32].

The induction of DNA double-strand breaks (DSBs) after irradiation of the developing mouse cortex has been explored by many groups over the years, by assessing different embryonic stages and postirradiation (PI) time points (summarized in Table 1). Irradiation-induced DSBs are immediate events that decrease between 1 h and 4 h after irradiation [31]. At E14.5, DSB foci are formed homogenously throughout the irradiated neocortex, with an equal distribution in the ventricular zone (VZ) and subventricular zone (SVZ), comprising stem and progenitor cells, and in the intermediate zone (IZ) and cortical plate (CP), comprising maturing neurons (Figure 2) [31]. Foci are observed in a linear dose-dependent manner and can be already detected after a low-dose of 0.01 Gy in the E13.5 irradiated cortex [33]. Interestingly, and in contrast to the appearance of DSB foci, DNA damage is not repaired at a similar speed in cells of the VZ/SVZ compared to CP cells. More specifically, in the mouse neocortex exposed to 2.0 Gy of *γ*-radiation, the decrease in DSB foci was much more pronounced in differentiating neurons than in VZ/SVZ cells between 1 h and 4 h PI, which was suggested to correlate with the high radiosensitivity of neuronal precursors [31]. Alternatively, this temporal difference in DNA repair might be better anticipated from the fact that both cell types utilize their own DDR machinery (see next section), in which repair occurs much faster in postmitotic neurons as compared to proliferating cells [34]. Differences in DNA repair kinetics depend not only on the cell type, but also on the administered radiation dose. This was shown* in vitro* using human cells [35] but was recently also demonstrated* in vivo* by Saha et al., who showed that, between 1 h and 6 h after irradiation, DNA repair did not take place in mouse neocortical cells exposed to doses below 0.05 Gy, while foci in 0.1-Gy exposed neocortices returned to background levels within this time span [33]. The implications of this finding dissociating the biological defense response between high and very low doses of radiation are not yet fully understood. It is however of particular interest in light of the increasing use of low-dose imaging modalities in current medical practice.

DNA damage repair requires dividing cells to arrest their cell cycle in order to activate the proper repair mechanisms, and this cell cycle block is again highly dependent on the administered radiation dose (see Table 1). Indeed, cell cycle arrest was induced in neocortical precursor cells exposed to 0.5 Gy, while 0.1 Gy was not sufficient to fully stop cell division and initiate DNA repair, probably because of an insensitivity to a low number of DSBs in such low-dose irradiated cells (Figure 2) [36, 47]. As a consequence of this escaped DNA repair, such cells survive and further differentiate, rendering them highly susceptible to persistent damage and ultimately cell death [36]. Whereas the intra-S and G2/M checkpoints were shown to be activated in neural progenitors after radiation, the G1/S checkpoint was not. This was proposed to result from the fact that p21, a key activator of G1/S cell cycle arrest, is not activated in neocortical stem cells. Contrarily, p21 was suggested to be activated in irradiated migrating cells of the IZ, where it is believed to have a proapoptotic role [38]. This finding is rather surprising, since a p53-dependent increase in p21 expression and a concomitant G1/S block were found in irradiated cultured neural progenitors by others [48, 49]. This discrepancy thus shows that we should always consider the influence of the extracellular environment and the specific structural organization of the neocortex that can only be recapitulated* in vivo*.

In all, we have gathered a basic understanding of radiation-induced DNA damage and repair over the past years, as summarized in Table 1, indicating that the DDR in the irradiated developing brain is not homogenously distributed but instead depends on the cell type and the administered radiation dose.

#### 3.1.2. Importance of DNA Damage Response Mechanisms for Normal Brain Development

The impact of a defective DDR in the embryonic brain is exemplified by the symptoms observed in patients with mutations in crucial DDR genes. These patients have a high risk of developing immunodeficiency, genomic instability, and cancer, but, surprisingly, they also frequently display neurological disorders, neurodegenerative diseases, or brain tumors [50–53]. Amongst those neurological disorders is microcephaly, a disease that is commonly evident at birth and very often observed in patients suffering from DDR-defective disorders [27]. One prominent example is microcephalin- (MCPH1-) dependent primary microcephaly, in which a defective ATR-dependent G2/M checkpoint arrest was evidenced [54, 55]. These observations prompted* in vitro* and* in vivo* research that disclosed substantial insight into the temporal use of DDR pathways, which is of vital importance during brain development to prevent an expansion of mutant progenitors and later occurring brain diseases [32, 53].

During the first days of neurogenesis, at which proliferation peaks, homologous recombination (HR) is the predominant DNA repair mechanism [56]. This was demonstrated by studies using Xrcc2-deficient mice, lacking the gene crucial for an efficient HR, in which a massive apoptosis of neural stem and progenitor cells between E10 and E14.5 results from unrepaired DNA damage [57]. On the other hand, studies using LigIV- and Xrcc4-deficient mice, which are characterized by impaired nonhomologous end-joining (NHEJ), unveiled the importance of NHEJ as a predominant repair modality during later phases of embryonic development [58–60]. In fact, in E14.5 LigIV null embryos, an induction of DSBs was observed in all cortical layers, to a similar extent as in 0.1-Gy irradiated control embryos (Table 1) [36]. In line with the clear time-dependency in repair mechanisms,* Rad54* ^−/−^ mice, lacking the crucial HR actor* Rad54*, were hypersensitive to ionizing radiation at the embryonic but not at the adult stage [61]. This demonstrates the importance of HR for the long-term survival of cortical progenitors irradiated during the S and G2 phases of the cell cycle, while it does not seem to be involved in a correct DNA damage repair of postmitotic neurons or progenitors in the G1 or G0 phase, when no sister chromatid is available to undergo HR [43].

As stated above, the adult brain is much less sensitive to radiation-induced damage in comparison to the developing brain, in which dividing cells show a higher susceptibility to DNA damage [29]. To note, astrocytes exhibit an even greater radioresistance than postmitotic neurons, as revealed by a strongly attenuated phosphorylation and reduced protein levels of ATM, CHK2, and p53, and a concomitant lack of apoptosis, even after an extremely high dose of 20 Gy [62]. Nevertheless, even though DNA repair is not that crucial as in dividing cells, neurons still need to survive from genotoxic stress since they are irreplaceable [63], which is also true for postmitotic neurons that reside in the CP of the neocortex. Differentiated cells remain capable of rejoining broken DNA ends through the collaboration of ATM and DNA-PK, or via nucleotide excision repair mechanisms in case of single strand breaks [64]. Even HR might still be used by cells that reenter the cell cycle upon genotoxic stress, which however predisposes the cells to die by apoptosis [65]. Whether these mechanisms also apply for mature cells in the developing cortex has however not been established yet.

#### 3.1.3. Radiation-Induced Neuronal Apoptosis

Radiation-induced neuronal apoptosis is well described in literature and is clearly evidenced in the developing neocortex (summarized in Table 1). A dose-dependent increase in apoptosis was found in the E14.5 cortex from a dose of 0.05 Gy onwards, with two to five DSBs being sufficient for a cell to initiate cell death [36]. Apoptosis is proposed to be the main outcome of unrepaired DNA DSBs in the developing nervous system, since dividing cells can be quickly replaced by the extensive neuronal stem cell pool [53]. With respect to the difference in radiosensitivity between proliferating and maturing neurons in the developing neocortex, radiation-induced apoptosis indeed seems to differ greatly between the VZ/SVZ and the IZ/CP. Possibly related to the slower repair kinetics of VZ/SVZ cells, these progenitors display the highest degree of apoptosis following radiation exposure [65]. Notably, however, the apoptotic response is believed to occur in two waves. Early after irradiation (3–6 h PI), apoptotic cells largely reside in the VZ/SVZ and IZ, with around 31% of all cells being apoptotic [31, 36]. At later time points (14 h and 24 h PI), a significant increase in apoptosis is noticeable in the IZ [36, 44] and CP [31, 39]. Whether apoptotic cells at this late stage are predominantly observed in these upper layers [39], or whether they are also still represented in the other proliferative layers [31], is however unclear. Either way, this second wave of apoptosis seems to contradict the general assumption of extensive cell death in proliferating cells. Yet it can be assumed that dying cells in the IZ/CP originate from proliferating cells with irreparable damage which progressed through mitosis and migrated to the upper neocortical layers where they undergo apoptosis, as was indeed suggested by Gatz et al. [36]. Intriguingly, apoptosis mechanisms and kinetics can also differ between different brain regions. This is for instance the case in the dorsal telencephalon, the site of origin for excitatory cortical neurons, and in the lateral ganglionic eminence (LGE), a source of striatal projection neurons and interneurons of the olfactory bulb and amygdala [66, 67]. In particular, in the LGE, intermediate progenitors of the SVZ were highly sensitive to radiation, whereas the radial stem cells were more resistant and entered self-renewal shortly after irradiation. This is in sharp contrast to the dorsal telencephalon in which the radial glia in the VZ are the main targets of early radiation-induced apoptosis [44]. This region-specific difference is likely correlated to a difference in the size of the SVZ, which is much more prominent in the LGE than in the dorsal telencephalon at the stage of irradiation. Mechanisms involved in this differential apoptotic response and functional consequences hereto are however not yet clarified [44]. Of note, it is also important to consider the large variation in parameters used by different authors to study radiation-induced apoptotic responses [31, 45]. For instance, differences in radiation dose, time points after exposure, and the delineation/definition of cellular layers all lead to a lack of unification of obtained results (see Table 1). In addition, the developmental stage at which radiation exposure takes place, as well as the irradiation parameters such as the dose-rate or energy of the beam, might significantly influence the apoptotic response. Nevertheless, radiation-induced apoptosis in the developing brain is highly dependent of p53 activation [42, 46], by the protein kinase ATM [68]. Phosphorylation of p53 and subsequent transactivation of apoptotic genes occur already at 2 h after irradiation [69]. However, p53-independent mechanisms also exist in the irradiated prenatal brain, which are able to induce cell cycle arrest but not apoptosis, as suggested from gene expression analysis of* Trp53*-null mice exposed to 0.5 Gy at E13 [70]. Unfortunately, a general consensus on which molecular players are involved in radiation-induced cell death in the developing brain is lacking. The participation of ATM and Bax in this radiation response was described both* in vivo* and* in vitro* [71, 72] but was disputed by another study [41]. Furthermore, caspase-9 [41] and caspase-3 [72] were defined as crucial death effectors in the central nervous system in response to irradiation, while this was contradicted by others [71, 73]. These opposing findings might be accredited to the use of a wide range of doses, including extremely high doses (≥10.0 Gy),* in vitro* set-ups, and irradiation at prenatal* versus* postnatal stages. A number of studies showed that radiation induces excitotoxic apoptosis, suggesting that N-methyl-D-aspartate (NMDA) receptor antagonists might be used as neuroprotectors after radiation exposure. This was for the first time investigated in the early 1990s for postnatal radiation injury, revealing an ameliorating effect of NMDA receptor blockage on radiation-induced hippocampal-dependent learning deficits [74] and neuronal damage [75]. More recently, it was shown that the NMDA receptor antagonist MK-801 could attenuate radiation-induced apoptosis in immature primary cortical neurons and embryonic brains [76]. However, other studies have reported an increased cell death in the mouse brain following postnatal delivery of MK-801 (reviewed in [77]), thus urging for further investigation into the mediators of these processes and the likelihood of excitotoxicity in the irradiated brain. Moreover, the administered dose of a particular NMDA receptor blocker needs to be carefully considered, especially with regard to incidental health risks posed to the pregnant mother. This concern is justified, since staggering and hyperlocomotion were detected in adult mice injected with doses of 0.3 mg/kg MK-801 and above [78, 79].

Despite the fact that the precise mechanisms remain unknown, it is generally accepted that radiation to the developing brain causes extensive apoptosis in a specific spatiotemporal manner. This radiation-induced apoptosis might contribute to the late occurring functional brain defects as observed in atomic bomb survivors, although the induction of early apoptotic events was never investigated in these cohorts. Therefore, to fully elucidate such a link, a more in-depth analysis of the causal relationship between short- and long-term consequences is warranted. This can for example be achieved by using transgenic animals lacking a radiation-induced apoptotic mediator in the developing brain and investigating possible changes in long-lasting brain defects.

#### 3.1.4. Radiation-Induced Defects in Neuronal Migration

Besides DNA damage, cell cycle arrest, and apoptosis, one other outcome of prenatal irradiation is the disturbance of neuronal migration. This is employed as a model system to induce cortical dysplasia in rats. More specifically, a dose of 2.25 Gy to the rat brain at E17 results in ectopic cell populations in the cerebral cortex and hippocampus, predisposing the animals to epileptic seizures, characteristic for the disease phenotype in human patients [80]. Also lower radiation doses were suggested to cause decelerated neuronal migration. For example, magnetic resonance imaging (MRI) of brains of mentally retarded atomic bomb survivors revealed large regions of abnormally situated gray matter, which the authors proposed resulted from defective migration around the timing of irradiation [5, 12]. Further evidence was gathered by Fushiki et al., who performed a BrdU pulse experiment in prenatally irradiated mice and discovered ectopic cells scattered over the SVZ, IZ, and CP instead of being restricted to the CP at 3 days following irradiation, which was clearly dose-dependent [81]. At early time points after birth, ectopic cells outside layer IV were still detected, whereas such cells were no longer observed at 8 weeks after birth, indicating that appropriate migration could be restored [81]. Similar migration defects in the embryonic brain were observed by others, with the severity depending on the dose [82–84]. Yet, of note, the observation of ectopic cells at later time points is only indirect evidence of disturbed neuronal migration. A first indication for an incorrect migration process was given by Sun et al. who showed that radial glia fibers, which serve as an essential substrate for outward neuronal migration in the dorsal neocortex [85], were disorganized, as defined by a distorted orientation and marked reduction in the number of these fibers (Figure 2) [83]. Still, a more detailed investigation at multiple early time points is desirable to determine if and how radiation hampers the migration route towards the CP. In addition, a time-lapse follow-up of these migrating cells would be extremely informative to unravel possible radiation-induced changes in migration kinetics.

#### 3.1.5. Radiation-Induced Microcephaly

The observation of microcephaly already within days after* in utero* radiation exposure of mice is believed to be largely attributable to the massive radiation-induced apoptosis (Figure 2) [86], but direct evidence linking the acute apoptosis with long-term brain anomalies is missing. As stated before, DDR pathways are fundamental to ensure a proliferative developmental nervous system, which is of importance after radiation exposure as well as in microcephalic disorders [27]. Unrepaired DNA damage and a resulting apoptosis or loss of proliferative capacity, for instance, induced by irradiation, can as such be the main underlying factor to induce microcephaly [27]. Reduction of cortical thickness was already revealed 24 h after 1.0 Gy exposure at E11 [39] and a decreased thickness of the postnatal cortex irradiated at E15 was discovered from a dose of 0.5 Gy onwards (Figure 2) [87]. One possible key factor underlying radiation-induced microcephaly is the abnormal spindle-like microcephaly associated gene (*ASPM*), which was downregulated in the ventricular zones 2 h after 2.0 Gy exposure to the E12 mouse brain [88]. The* ASPM* gene was convincingly linked to microcephaly, as a homozygous mutation of this gene is responsible for autosomal recessive primary microcephaly (*MCPH*) in humans [89]. Together with a downregulation of* ASPM*, the induction of supernumerary centrosomes due to centrosomal overduplication was also described to result from radiation exposure, albeit only shown in human tumor cells after high irradiation doses [90]. Such centrosomal deficiencies are often observed in cells of patients with primary microcephaly, where they contribute to cell death or an aberrant proliferation [90]. Microcephaly is also often related to mitotic spindle defects, as described for MCPH where spindle defects are the result of mutations in, for example, MCPH1, WD Repeat Domain 62 (WDR62), CDK5 Regulatory Subunit-associated Protein 2, and ASPM (for an overview, see [91]). Here, mitotic spindle defects lead to a perturbation of the intricate balance between symmetric and asymmetric divisions, in favor of asymmetric cell divisions and thus of a premature differentiation, leading to a notable reduction in the number of neural precursors and ultimately in overall brain size [92]. In light of the microcephalic phenotype in radiation-exposed brains, and the reduction in ASPM expression in irradiated brains as described earlier [88], it would thus be meaningful to investigate the possibility of a premature differentiation in prenatally irradiated brains, which has not been studied in much detail before. Recently, however, a thorough gene expression analysis suggested that* in utero* irradiation triggers a p53-dependent induction of genes associated with neuronal differentiation and mitotic spindle assembly [69], hinting for a possible premature differentiation following radiation exposure. This hypothesis was further strengthened by the very strong overlap between gene expression profiles of irradiated embryonic brains and those of Magoh^+/−^ mice, which display microcephaly associated with premature neuronal differentiation [69, 93]. A follow-up of these mice showed that apoptosis, but not premature neuronal differentiation, was p53-dependent [94]. A more detailed investigation should thus be performed to assess whether this also applies to irradiated mouse embryos.

In all, microcephaly as a result of prenatal irradiation is starting to be further explored, with a growing awareness of similarities between radiation-induced and microcephaly disease genes that might converge to related mechanisms. Yet* in vivo* evidence for radiation-induced mitotic spindle defects and premature differentiation remains to be provided, for example, via investigation of the division angle in neocortical stem cells.

### 3.2. Long-Lasting Structural and Functional Effects

#### 3.2.1. Depletion of Cells in the* In Utero* Irradiated Brain?

The prenatal radiation-induced microcephaly, as established both in humans and in animals, is mostly accompanied by overall growth retardation [95]. This effect appears to be induced from a dose of 0.3 Gy on [95]. Whether the reduction in brain size is associated with an overall decrease in the number or density of neurons remains however disputed. The majority of animal studies are in agreement with a reduced cell number, for instance, evidenced for the E15 irradiated rat brain by means of MRI analyses and histology [96, 97], and further substantiated for the irradiated rodent hippocampus, corpus callosum, cerebellar Purkinje cells, and primary visual cortex [98–103]. Contrarily, other studies found no differences in cell number in the somatosensory cortex, in cortical layer V pyramidal neurons [104, 105], and in the visual cortex [105] after irradiation, the latter being in sharp contrast with the study of Vitral et al. who showed a strong neural reduction in the irradiated visual cortex [100]. Interestingly, a change in cell density can strongly depend on the cell type within a single brain region, as shown for the irradiated cerebellum. Here, stereological investigations revealed a reduced Purkinje cell number but an increased granule cell number after low-dose irradiation of the E13 embryo [106]. The authors reasoned that this seeming paradox might be due to an increased granule cell production after radiation-induced ROS production but also highlighted the crucial importance of timing of radiation exposure, given that both cell types were decreased in number after low-dose exposure at later stages of development (E17–E19) [107].

Apart from rodents, several studies on prenatally irradiated nonhuman primates have been performed in which consecutive developmental events are more spread over time, as compared to the overlapping sequence of events in the short gestational period of rodents. Therefore, such data provided a better insight into the importance of developmental timing to develop irradiation-induced defects. Specific depletion of cells born at the stage of irradiation was found and consequent volume reductions occurred only in the regions and/or layers constituted by those cells, after irradiation of macaques during early- or midgestation [108–111]. Such a straightforward relationship was indeed not always evident in rodent studies. For example, irradiation of the E11 mouse embryo, a stage at which cortical layer VI cells are being produced, caused a decrease in the thickness of layers I, II, and III. Further, irradiation at E13, corresponding to formation of cells that will constitute layer V, resulted in a decrease in layers IV and VI cells. Finally, irradiation at E15 or E17 surprisingly affected the thickness of all cortical layers [112]. This finding is puzzling and implies that irradiation of progenitors during a specific developmental time-window might not only impact cells typical for its birth date, but instead might disrupt a more elaborate neuronal network and cytoarchitecture. Over the years, different hypotheses have been proposed to explain cortical diversity. One of those, called the “common progenitor hypothesis,” claims that the fate of a common progenitor changes over time to generate the different subtypes of neurons. This thus implies that early progenitors, normally producing lower layer neurons, are also capable of producing upper layer neurons (reviewed in [113]), which explains to some extent our findings after prenatal radiation exposure. On top of that, this suggests that irradiation during an early developmental period has the greatest impact on cortical layer formation. This hypothesis would indeed favor the finding of a hampered juvenile hippocampal neurogenesis resulting from acute irradiation at the onset of neurogenesis [39], since hippocampal neurons start to be produced at an early stage of E10.5, followed by a peak of pyramidal neuron production only several days later [114–116].

#### 3.2.2. A Disturbed Neural Circuit Formation after Prenatal Irradiation

As mentioned before, the observed disruption of neuronal migration following irradiation causes the introduction of ectopic cells spread throughout the brain [117–120]. Such a disorganization of neurons can be accompanied by a defective neuronal orientation, morphology and arborization, resulting from an improper and disturbed maturation, as was suggested for irradiated corticospinal tract neurons [121], CA1 neurons [122], pyramidal cells of cortical layer V [123], and hippocampal mossy fibers [124]. Evidently, such a disturbed dendritic organization might also entail an improper neural circuit formation and synaptic communication. Indeed, a decreased inhibition of excitatory cells was shown in the cortical dysplasia model, probably contributing to the epileptogenic development in these animals [125]. Likewise, an increase in excitatory synapse markers in specific layers of the cortex [126] and an elevated glutamate release [127] were demonstrated after radiation-induced cortical dysplasia. In neurons cultured from irradiated neocortices, a disturbed synaptic communication was further unveiled by an increased inhibitory* versus* excitatory input [128]. Yet a firm conclusion on the effects of radiation on synaptogenesis is hindered by the lack of evidence for the low-dose range and by many contradictory findings between studies. For example, no difference in the number of synapses was found in layer V cortical cells of the E15 embryo irradiated with different doses [123], and synapse density was normal in the somatosensory and visual cortex after 1.0 Gy exposure [105]. On the other hand, a proteomic study on hippocampal samples from 6-month-old prenatally irradiated mice revealed an enhanced expression of postsynaptic density protein 95 (PSD95) after 1.0 Gy exposure, suggesting a pronounced effect of moderate doses of irradiation on synaptic plasticity in hippocampal dendrites [129].

Thus, these findings demonstrate the necessity to further explore neuronal communication after prenatal irradiation and to investigate synaptogenesis and inhibitory neuron development at multiple time points following irradiation, using a broad range of irradiation doses.

#### 3.2.3. Brain Structure and Function Deficits after Prenatal Irradiation

At high doses of exposure (≥1.0 Gy), the dispersed cell masses supposedly associated with a defective migration are accompanied by large cytoarchitectural aberrations [80]. As an illustration, ectopic gray matter was found at the ventricular boundaries in adult animals prenatally exposed to radiation, indicative of cells that have not migrated at all since development [130]. Moreover, the typical 6-layered cortical architecture and the hippocampal laminar formation of CA1, CA3, and/or DG often appear severely impaired after high-dose prenatal irradiation [131, 132]. Notably, these high doses have been shown to produce such a large spectrum of defects in the postnatal, juvenile, and/or (young) adult brain, with structural changes that completely disrupt the brain's integrity. Examples for this are the underdevelopment or even complete loss of white matter structures such as the corpus callosum [100, 133], severe cerebellar deficits [99, 103], and extreme hydrocephalus [134, 135]. As such, it is not surprising that animals irradiated with doses ≥1.0 Gy display a severely affected behavior. In the following paragraph and in line with our focus on low to moderate doses of irradiation, we will further report on mild changes that are induced by this lower dose range, unless findings in which higher doses were used can be informative to explain underlying mechanisms.

The use of behavioral tests to assess brain function after* in utero* irradiation is frequent and is considered as the best available strategy to translate animal behavior to neurological changes seen in the atomic bomb survivors. Ample evidence from rodent studies exists regarding alterations in activity, locomotor, exploratory, and anxiety-related behavior, as well as in cognition for doses ≤1.0 Gy [95, 136–140]. Recently, a proteomics study uncovered changes in several signaling pathways, 6 months after 0.5 and 1.0 Gy exposure at E11, which correlate well with these cognitive defects [129]. However, likely resulting from interlaboratory variation in behavioral testing, but probably also due to differences in radiation doses and dose-rates used, many discrepancies exist between studies. In particular, no consistent conclusions can be drawn regarding the gestational age at which rodent behavior is mostly affected by irradiation. For example, 0.35-Gy and 1.0-Gy exposure to the E11-E11.5 mouse embryo induced changes in locomotor and anxiolytic behavior as well as in learning and memory, while no changes were observed when exposure occurred at earlier or later stages [39, 141]. On the other hand, exposure to an intermediate dose of 0.5 Gy surprisingly resulted in behavioral alterations for all gestational ages [141]. Furthermore, Sienkiewicz et al. postulated that the E15 and E18 embryo was most sensitive to learning and memory deficits after 1.0 Gy exposure, when compared to embryos irradiated at E13, although only a limited number of tests were performed in this study [142]. Thus, it is clear that the exact functional deficits resulting from prenatal irradiation are far from completely understood and that additional research is required, preferably using the same behavioral test battery and similar radiation types and dose range for each gestational stage. Nevertheless, many research groups appear to observe a threshold dose below which no behavioral changes can be detected. This threshold is believed to be around 0.3 Gy in mice [95, 112, 138, 139]. Noteworthy, in humans, a total dose of 0.2 Gy is regarded as a threshold value above which a therapeutic abortion should be considered [143], while a fetal dose not exceeding 0.1 Gy and preferably 0.05 Gy is strongly advised [144, 145]. This might relate to a possible higher radiosensitivity of humans as compared to rodents but might also indicate a lack of sensitive protocols to assess functional deficits in prenatally irradiated animal models.

Other alterations that have been observed and that might contribute to persistent structural and functional deficits after* in utero* radiation exposure are, for instance, inflammation and vascular modifications. Irradiation of rats at E11 with 1.3 Gy or at E15 with 1.5 Gy was shown to induce astrogliosis and astrocyte proliferation in the hindbrain [146] and in the whole brain [96], respectively. In the context of vascular modifications, arterial defects were discovered in E13 mouse brains after 0.5 Gy irradiation [135]. Furthermore, a dose of 1.5 Gy resulted in an underdevelopment of the microvasculature, responsible for a decreased cerebral blood flow and angioarchitectonic abnormalities. However, the BBB and blood-cerebrospinal fluid barrier were not altered [147]. Of note, most research on radiation-induced BBB permeability has been focused on high doses, in the context of radiotherapy research where an increased permeability is desirable for the delivery of chemotherapeutics to the brain [148]. As such, due to the poor amount of data, effects on BBB permeability after lower doses of irradiation might be overlooked and should be further explored. Besides, since the blood-brain barrier is still immature in the developing embryo and more prone to drugs, toxins, and pathological conditions [149], special attention should be directed to effects of prenatal irradiation on BBB formation and associated neurological disorders in later life.

In conclusion, brain functional and structural modifications following prenatal irradiation are far-reaching. Yet, despite small brain size and ectopic neurons, there is no consensus about the exact brain defects or their link to the observed behavioral changes. Then again, such a link might be difficult to establish, given that higher doses of irradiation generate such widespread changes. Therefore, we suggest that the use of low doses, which is becoming extremely relevant in the medical sector, needs to be exploited further to detect more subtle effects on brain structure and function and to provide as such a better model to study underlying mechanisms and possible causative relationships.

#### 3.2.4. Prenatal Radiation-Induced Senescence and Ageing?

To our knowledge, the influence of prenatal irradiation on ageing and in particular brain ageing has never been studied. Nonetheless, evidence exists to suggest that prenatal radiation might aggravate or at least influence to a certain extent ageing processes in the brain. This can be suggested by the fact that early-life DNA damage and replicative stress are believed to control the onset of ageing [150–152] and by the fact that the developing embryonic brain is by far the most sensitive organ to DNA damage events [27, 153]. Indeed, as described in previous paragraphs, irradiation induces a substantial amount of DNA damage in the embryonic neocortex, stressing the predisposed risk of accelerated ageing. The link between genomic instability and ageing is further corroborated by the accelerated ageing or neurodegeneration observed in patients and mice with defective DNA repair mechanisms [51–53, 154]. Although most of these models display chronic DNA damage, which is in contrast to acutely irradiated mice, a mouse model for ATR-Seckel syndrome displayed accelerated ageing despite DNA damage occurring only during embryonic development [150]. Other underlying pathways for a possible radiation-induced premature ageing might involve changes in tau expression, as recently demonstrated in primary cultured hippocampal cells exposed to 0.5 and 2 Gy [155]. Still, a clear-cut answer as to whether senescence can be a mechanism that contributes to radiation effects in the developing brain is unknown. In favor of such a link, an induction of senescence/ageing markers could be revealed in various tissues after adult mouse irradiation, including DNA damage foci, p21, and senescence-associated *β*-galactosidase expression and the mitochondrial common deletion [156, 157]. Also in adult brain tissue, albeit only after high radiation doses, ageing-like processes such as oxidative stress and inflammation were found to occur [158–161]. Finally, some prenatal radiation-induced alterations in behavior were investigated at multiple time points and worsened over time, indicative of a progressive decrease in brain function [162]. This was also observed in nonhuman primates, in which cognitive impairment after* in utero* irradiation was not yet evident in young adulthood but did manifest at an adult age [163].

All the above suggests that taking a closer look at* in utero* radiation-induced ageing might be very useful, particularly with respect to the increased use of low-dose exposures and the steady growth of the elderly population. In first instance, investigation of ageing parameters as has been done for adult high-dose irradiation should be performed, substantiated by an examination of cognition and brain functionality at the long term. Atomic bomb survivors have now reached about 70 years of age and the follow-up of their health might already provide crucial information in this regard. Interestingly, an accelerated occurrence of age-related changes, modelled in terms of cardiopulmonary and psychological age, was indeed shown in clean-up workers of the Chernobyl area [164]. Unfortunately, no scientific consensus can be found in the atomic bomb survivor life-span study, due to an insufficient statistical power [165] and a biased dose-mortality relationship caused by an incomplete knowledge on received doses [166].

## 4. Implications for Radiation Protection

Although the precise consequences of* in utero* radiation exposure are not yet fully understood, the risk for the unborn child to develop brain abnormalities following exposure is generally recognized. Guidelines for pregnant women needing medical intervention using radiation currently apply the ALARA (As Low As Reasonably Achievable) principle, and the exposure dose should not exceed 0.1 Gy [144, 145], as implemented by the ICRP. This threshold implies that no noncancer effects are expected to occur below 0.1 Gy and that the increased risk to develop cancers is neglectable. However, the proposed sharp threshold is due to many uncertainties that still exist on the exact consequences of prenatal radiation exposure, warranting a better understanding of the broad range of radiation effects to improve the outcome for pregnant women and to better inform medical professionals. This is especially important given that the current use of high but especially of low-dose radiation rises continuously in the clinic. For example, in the United States, the collective radiation dose from medical imaging purposes has increased no less than 6 times over the last two decades [167]. Notably, whereas a single CT scan is not believed to administer a dose that can provoke a negative outcome to the patient, it is the cumulative dose from multiple scanning sessions and the unjustified use of scans that might pose a considerable risk to human health [3, 4, 168]. In case of exposure to the embryo/fetus, evidence indicates an increased cancer risk for doses as low as 6 mGy [169], which is in the range of those received by some CT examinations (see Table 2 for an overview of doses received after various radiation types).

## 5. Conclusions

Ever since its discovery, ionizing radiation has proven to be a double-edged sword [177]. Its use in the medical field is of crucial importance, but it must be utilized with great caution since exposure to radiation might evoke serious health consequences, as pointed out for* in utero* exposure in this work. Unfortunately, a detailed view on short- and long-term brain defects following prenatal exposure is incomplete, which is why it is necessary to integrate more animal studies, more radiation doses, and a broader range of developmental time points to be considered. Indeed, epidemiological studies do not allow identifying subclinical health effects that can develop into more serious health risks later in life. In light of such long-lasting functional brain defects, a recent study argued that little information exists on the long-term outcome of radiotherapy-exposed fetuses [145], for which the use of a good mouse model might hold the key to better understand such a correlation and to achieve a correct health risk assessment. Another uncertainty is the effect of low-dose irradiation and whether it would fit in a linear-nonthreshold (LNT) model [178] or would rather induce a hormetic, or an aggravated, health status. In fact, estimates of low-dose risks are too often an extrapolation from high doses, while it is important to realize that low-dose risks should be based on a sufficient knowledge on the underlying biological mechanisms. In this respect, evidence is culminating over the years which disputes a LNT relationship for low radiation doses, in terms of both carcinogenic and noncarcinogenic effects [179–182].

Taken together, the growing awareness of radiation defects in the developing brain is an important step forward in a complete understanding of early and persistent brain defects occurring after* in utero* radiation exposure, which will help to improve the health care of expecting mothers and their unborn children after exposure to various types of radiation sources.

## Figures and Tables

**Table 1:** DNA damage, repair mechanisms, and cell death in the irradiated prenatal neocortex of rodents.

Stage	Time after irradiation	Dose	Effect	References
			*DNA damage and repair*	

E13.5	1 h	0.01–0.1 Gy	Dose-dependent DNA damage	[33]
	6 h	0.1 Gy	Strong reduction in DNA damage foci, not observed at lower doses (0.01–0.05 Gy)	[33]

E14.5	1 h	0.1–0.5 Gy	Dose-dependent and widespread DNA damage, average of 2-3 foci/cell	[36, 37]
			NHEJ in VZ/SVZ 0.1 Gy: partial G2/M cell cycle arrest, 4–6 DSBs in G2 cells 0.5 Gy: full G2/M arrest	[36]
		2.0 Gy	Widespread DNA damage	[31]
	4 h	2.0 Gy	G2/M checkpoint release/restart of mitosis	[38]
	6 h	0.5 Gy	G2/M checkpoint release with 67% of mitotic cells having remaining DSB foci	[36]
	24 h	2.0 Gy	Full DNA damage repair, which occurred slower in VZ/SVZ progenitors	[31]

			*Apoptosis*	

E11	24 h	1.0 Gy	Apoptosis mainly restricted to CP	[39]

E12.5	4 h	14 Gy	Widespread apoptosis, ATM-independent	[40]

E12–E13	6 h	1.0 Gy	50% decrease in viable cells, caspase-9 dependent	[41]

E13-E13.5	6 h	0.01–0.2 Gy	Increased apoptosis mainly restricted to VZ/SVZ	[33]
		1.5–3 Gy	Dose-dependent, widespread apoptosis	[42]

E14.5	4–8 h	1.0–2.0 Gy	Dose-dependent increase in apoptosis, gradual from VZ (high) to IZ (low) Rad54 (HR)-dependent cell survival in S- and G2-phase irradiated cells	[38, 43]
	3-4 h	2.0 Gy	High radiosensitivity of G2/M phase cells, p21-independent Proapoptotic effect of p21 on IZ cells Cells irradiated in S-phase enter intra-S checkpoints, leading to delayed INM and apoptosis	[38]
	6 h	0.05–0.1 Gy	Moderate apoptosis mainly in VZ/SVZ	[36]
		0.5 Gy	>1% apoptosis, mainly in VZ/SVZ 75% ATM-dependent in VZ/SVZ, 15% ATM-dependent in IZ/CP	[36]
	14 h	≥0.1 Gy	Reduced apoptosis in VZ/SVZ Apoptosis in IZ resulting from persisting DSBs	[36]
	24 h	2.0 Gy	Peak of apoptosis still in VZ/SVZ, but increased apoptosis in CP	[31]
			Peak of apoptosis in the IZ	[44]

E15-E15.5	3 h	0.5 Gy	60% apoptosis in SVZ, 25% in IZ + CP	[45]
	4 h	14 Gy	Widespread apoptosis, ATM dependency only in SVZ	[40]
	24 h	0.5 Gy	Gradual increase in upper layer apoptosis; 70% apoptosis in IZ + CP	[45]

E17	4 h + 24 h	0.1–0.2 Gy	Apoptosis in VZ/SVZ + IZ, no difference in PI time points	[46]
		0.4 Gy	Apoptosis in VZ/SVZ + IZ + CP, no difference in PI time points	[46]

E = embryonic stage, Gy = gray, NHEJ = nonhomologous end-joining, HR = homologous recombination, DSB = double-strand break, VZ = ventricular zone, SVZ = subventricular zone, IZ = intermediate zone, CP = cortical plate, ATM = ataxia telangiectasia mutated, and INM = interkinetic nuclear migration.

**Table 2:** Societal relevant radiation doses.

Type of irradiation	Dose (mSv)
One return flight New York-London	0.10
Typical dose of one year on the ISS	170
Mammography	3
Radiography: chest	0.10
Radiography: abdomen	1.2
CT: head	2.0
CT: abdomen	6–10
CT: pelvis	8–10
Radiotherapy (fractionated)	40.000–70.000

	Mean fetal dose (mGy)

Radiography: chest	<0.01
Radiography: abdomen	1.4
CT: head	<0.005
CT: abdomen	8
CT: pelvis	25
Breast cancer radiotherapy (50 Gy to the mother)	50–150

Doses are whole-body doses, except for those of medical exposure, which are delivered to a specific organ or the embryo/fetus. CT: computed tomography, Gy: gray, ISS: international space station, Sv: sievert. Based on [167, 170–176].
